# Improved Neurological Outcome of Perampanel for Hypoxic-Ischemic Encephalopathy in Patients After Out-of-Hospital Cardiac Arrest Resuscitation

**DOI:** 10.7759/cureus.51392

**Published:** 2023-12-31

**Authors:** Yoshimi Nakamichi, Ryo Ichibayashi, Masayuki Watanabe, Ginga Suzuki, Hibiki Serizawa, Saki Yamamoto, Yuka Masuyama, Mitsuru Honda

**Affiliations:** 1 Emergency Medicine, Toho University Medical Center Omori Hospital, Tokyo, JPN; 2 Internal Medicine, Toho University Medical Center Sakura Hospital, Chiba, JPN

**Keywords:** neurological outcome, electroencephalogram monitoring, automated external defibrillator, hypoxic-ischemic encephalopathy, post-resuscitation care, out-of-hospital cardiac arrest

## Abstract

Background: Although the resuscitation rate for out-of-hospital cardiac arrest (OHCA) patients in Japan is increasing due to the widespread use of automated external defibrillators, the proportion of patients who can return to society remains low at approximately 7%. Many patients have poor neurological outcomes and cannot return to society because of post-resuscitation hypoxic-ischemic encephalopathy. While the resumption of cardiac rhythm is important for patients with OHCA, improving neurological outcomes and returning to society are also important.

Objectives: To investigate whether perampanel, an antiepileptic drug that provides neurological protection against stroke and head injury, could improve neurological outcomes in patients resuscitated after OHCA.

Methods: The participants included 33 patients with OHCA admitted to our hospital from January 2021 to June 2022 and 33 patients admitted before that time. Perampanel was administered to the patients in the intervention group immediately after resuscitation. We defined a *Cerebral Performance Category *(CPC)* score* of 1.2 as a good neurological outcome.

Results: There was no significant difference in neurological outcomes at intensive care unit discharge between the intervention and non-intervention groups (number of CPC 1.2: 16/33 vs. 9/33); however, neurological outcomes at hospital discharge were significantly better in the intervention group (number of CPC 1.2: 19/33 vs. 9/33 P = 0.01).

Conclusion: The α-amino-3-hydroxyl-5-methyl-4-isoxazole-propionate receptor inhibitory and neuronal protective effects of perampanel may have inhibited the progression of hypoxic-ischemic encephalopathy, which develops after the resumption of cardiac rhythm, and suppressed neuronal damage. Early administration of perampanel after resuscitation of patients with OHCA may improve neurological outcomes.

## Introduction

Although the resuscitation rate of patients with out-of-hospital cardiac arrest (OHCA) in Japan has been increasing because of the widespread use of automated external defibrillators (AEDs), according to the Fire and Disaster Management Agency of the Ministry of Internal Affairs and Communications in Japan, only approximately 7% of these patients can return to society and resume their normal lives [1]. In many cases, the central nervous system is damaged due to hypoxic-ischemic encephalopathy resulting from cardiac arrest, and even if the patient is successfully resuscitated, the lack of improvement in neurological outcomes makes it impossible for the patient to return to society and daily life [2]. Recently, temperature management therapy has been introduced to improve neurological outcomes. However, there is a wide range of opinions and disagreements regarding temperature setting and duration [3-5]. There is no effective treatment for hypoxic-ischemic encephalopathy. Perampanel, an antiepileptic drug, acts in an inhibitory manner on α-amino-3-hydroxyl-5-methyl-4-isoxazole-propionate (AMPA) receptors in the excitatory nervous system and acts noncompetitively with glutamate to produce excitatory inhibition and antiepileptic effects [6]. In animal studies, perampanel has been reported to protect neurons during cerebral infarction and severe traumatic brain injury [7]. We investigated the effects of early perampanel administration on neurological outcomes in patients with OHCA after resuscitation.

This article was previously posted to the Research Square preprint server on September 9, 2023.

## Materials and methods

Participants

This study included 34 consecutive patients with endogenous OHCA who were admitted to the critical care center of a university hospital from January 2021 to June 2022. The comparison group consisted of 33 consecutive patients with endogenous OHCA who were admitted to the critical care center of a university hospital between January 2019 and December 2021 and treated intensively after the return of spontaneous circulation (ROSC). Patients in cardiopulmonary arrest at the time of hospital admission had a Glasgow Coma Scale (GCS) score of ≤8 after ROSC. Alternatively, patients who had ROSC outside the hospital but had a GCS score of ≤8 at the time of admission were also included in the study.

The protocol for this research project was approved by our hospital's institutional ethics committee, and it conforms to the provisions of the Declaration of Helsinki. Informed consent was obtained from the patient's family and surrogate since it is difficult to obtain informed consent from a patient undergoing intensive care after ROSC.

Exclusion criteria

Patients with unclear corticomedullary borders or loss of brain sulci on CT scan after ROSC, patients with end-stage malignant disease, patients younger than 15 years of age, patients with ROSC with family members who do not want intensive care, and patients with ROSC and primary disease that cannot be resolved were excluded from this study.

Comparison item

Neurological outcomes at intensive care unit (ICU) discharge, neurological outcomes at hospital discharge, length of ICU stay, length of hospital stay, ICU mortality, overall mortality, and electroencephalogram (EEG) findings were compared.

Resuscitation procedure

Standard advanced cardiovascular life support was performed. Extracorporeal cardiopulmonary resuscitation (ECPR) was performed when cardiogenesis was suspected. The decision to perform ECPR was made at the discretion of the primary care physician.

Method of administration of Perampanel

Perampanel (4 mg) was administered through a gastric or duodenal tube immediately (within 12 h) after admission to our ICU. Administration of perampanel was terminated when an improvement in the level of consciousness was observed; however, it was used for 14 days if no improvement was observed. Following the observation of epilepsy or epileptic electroencephalograms, the drug was continued as needed as an antiepileptic drug when deemed necessary.

ICU management

Both groups were managed similarly except for perampanel administration. In our hospital, patients with cardiopulmonary arrest have successfully undergone ROSC treatment for the causative disease. The patient was then placed in the ICU for post-resuscitation under deep sedation. The patient's condition was maintained; however, additional treatment was administered when necessary. Target temperature management (TTM) maintenance therapy was introduced 72 hours after resuscitation. The target temperature was 36 ℃ or 34 ℃. In the case of 36 ℃, the patient was maintained at 36 ℃ for 3 days; in the case of 34 ℃, 34 ℃ was reached immediately after ROSC, followed by 24 h of maintenance and 48 h of rewarming. If bleeding or other problems were present, the temperature was maintained at 36 °C. Although the temperature setting is often left to the judgment of the initial responding physician, the post-cardiac arrest syndrome for induced therapeutic hypothermia (rCAST) [8] is used as a predictive score for neurological outcome after ROSC, and a target body temperature is set.

Electroencephalography (EEG)

The first EEG was performed within 24 hours of ICU admission. Because hypoxic-ischemic encephalopathy was fixed 48-72 hours after resuscitation, sedation was terminated 72 hours after resuscitation, and a second EEG examination was performed to evaluate neurological outcomes. After that, EEG examinations were performed if the level of consciousness did not improve or for other reasons.

Endpoints

The CPC classification score was used. The group with a CPC score of 1-2 was the good outcome group, and the other was the poor outcome group. We compared the CPC scores in both groups at ICU and hospital discharge.

Statistical methods

Easy R version 1.61 (Jichi Medical University Saitama Medical Center, Saitama, Japan)[9] was used for all statistical analyses, and the χ2 independence test (chi-squared test) was used to compare the proportions of categorical variables. The median and interquartile range were used for continuous variables, and the Mann-Whitney U test was used for comparisons, with statistical significance set at P <0.05.

## Results

Excluding one patient in the intervention group who had a GCS score of ≤8 at presentation, which improved immediately to a GCS score of 11 after ROSC, 33 patients in the intervention group received perampanel immediately (within 12 h) after ROSC and 33 in the non-intervention group. The background of the conditions causing cardiopulmonary arrest in both groups and the background of admission are shown in Table 1. ECPR was performed in 15 cases in the intervention group and 19 cases in the non-intervention group, with no significant intergroup difference. There was no difference in the number of cases in which TTM was introduced after ROSC between the two groups, and there was no significant difference in the number of cases in which TTM at 34℃ was performed (21 in the intervention group and 19 in the non-intervention group) (Table 1). No significant differences were observed in the presence or absence of witnesses/bystanders. There were no differences in prehospital defibrillation, either in the use of AEDs by the general public, the number of defibrillations performed by the emergency medical services (EMS) team, or the total number of defibrillations performed by both groups. The EMS team only administered prehospital adrenaline in cases of non-defibrillation indications; however, there was no difference between the two groups. The cardiac arrest and ROSC times were also similar between the two groups.

**Table 1:** Characteristics of Patients ECPR: extracorporeal cardiopulmonary resuscitation; TTM: target temperature management; rCAST: post-Cardiac Arrest Syndrome for induced Therapeutic hypothermia; PER: Perampanel; CPR: CardioPulmonary Resuscitation; AED: Automated External Defibrillator; GCS: Glasgow coma scale

	PER＋(n=33)	PER－(n=33)	p-value
Demographic characteristics			
sex(number of male)	26	26	1
age	57.2±12.7	61.2±17.3	0.08
Bystander CPR (number)	24	16	0.08
Witness（number)	24	23	1
pre-hospital treatment			
Defibrillation			
emergency medical services (times)	1.36±1.56	1.24±1.28	1
AED by bystander (times)	0.42±0.83	0.42±0.97	0.67
total	1.75±1.62	1.67±1.47	0.81
Adrenaline administration (㎎)	0.27±0.71	0.39±0.93	0.7
Hospital treatment			
Defibrillation (times)	0.3±0.9	0.1±0.2	0.13
adrenaline administration (㎎)	0.5±1.1	0.5±0.8	0.77
ECPR (number)	15	19	0.46
TTM (number)	31	29	0.67
TTM (34.0℃) (number)	21	19	0.81
Monitored rhythm			
First			
Shockable (number)	22	21	1
Hospital			
Shockable (number)	10	11	1
Estimated cardiac arrest time to return of spontaneous circulation (min)	33.1±16.7	32.8±12.5	0.7
return of spontaneous curculation at out of hospital (number)	15	9	0.2
Post-resuscitation GCS	3.39±1.12	3.27±1.15	0.27
rCAST score	10.9±４	12.3±4.1	0.1
Characteristic of cardiac arrest			
cardiac	23	25	0.78
other	6	6	1
unknown	4	2	0.4

The rCAST score was used to assess the severity of illness at ROSC and compare the pre-admission status; however, there was no significant difference between the intervention and non-intervention groups (P = 0.1). Initial electrocardiograms (ECGs) at cardiopulmonary arrest were classified as shockable and non-shockable and were compared in both groups; however, there were no significant differences. No significant differences were observed in the causes of cardiac arrest.

The two groups had no significant differences regarding the results of blood samples taken at admission related to neurological outcome [10-12], including pH, potassium levels, glucose, base excess, anion GAP, partial pressure of carbon dioxide in arterial blood (PaCO2), and ammonia levels. No significant differences in patient background, disease status, or condition between the two groups were observed. In addition, a report [13,14] shows that the lower the gray-white-matter ratio (GWR) on head CT at admission, the poorer the neurological outcome. A report also uses this ratio to indicate neurological outcome, so GWR was compared in both groups; however, there was no significant difference (Table 2).

**Table 2:** Laboratory data PER: Perampanel; LAC: lactic acid; CO_2_: carbon dioxide; HCO_3_: hydrogencarbonate; GLU: glucose; K: Potassium; NH_3_: ammonia; CT: computed tomography; GWR: gray-to-white matter ratio

	PER+(n=33)	PER-(n=33)	p-value
pH	7.1±0.2	7.1±2.5	0.31
LAC (mmol)	10.8±4.9	11.8±6.3	0.69
CO_2_(mmHg)	60.6±25.5	68.3±32.8	0.35
HCO_3_(mEq/L)	20.2±14.0	18.3±5.9	0.97
GLU(g/dl)	283.9±92.4	279.3±102.4	0.81
Base Excess(mEq/L)	-12.1±8.3	-12.8±9.4	0.47
Anion Gap	8.25±8.2	9.6±6.6	0.39
K(meq)	3.8±1.0	4.0±0.9	0.39
NH3(μg/dl)	135±110	177±237	1
CTgrade			
GWR	1.180±0.048	1.177±0.048	0.72

At ICU discharge, 48% (16 patients) of patients in the intervention group and 27% (9 patients) in the non-intervention group had a CPC score of 1.2, with no significant difference between the two groups (P = 0.12). The outcome at hospital discharge was significantly better in 58% (19 patients) of patients in the intervention group and 27% (9 patients) in the non-intervention group (P = 0.01).

Three patients in the intervention group showed improvement in their level of consciousness after leaving the ICU, including one who recovered from a CPC score of 4 to a CPC score of 2 and another patient who was in a coma for a month; however, the patient's consciousness level improved. The patient was transferred to a rehabilitation hospital, where the patient eventually had a good outcome. However, none of the patients in the non-intervention group had improved CPC scores at hospital discharge compared to ICU discharge.

Thirty-one patients in the intervention group and 31 in the non-intervention group who underwent EEG testing were compared. The EEG was classified as highly malignant EEG (HMEEG) for suppressed background, suppressed background with continuous periodic discharges, and burst-suppression background. Other EEGs were classified as non-highly malignant EEGs (NHM) [15,16]. The first EEG test was performed within 24 hours of admission. The HMEEG group included 19 patients. Comparing the good outcome results of the EEG in both groups, 9 of the 19 patients in the intervention group with good outcomes (CPC score 1 or 2 at discharge) had HM findings on the initial EEG; in contrast, none of the patients in the non-intervention group with good outcomes had HMEEG. Although there was no significant difference (P = 0.06), there was a trend toward more HMEEG findings in the good outcome intervention group.

There was no significant difference in ICU mortality between the two groups (p=0.42); however, there was a trend toward a higher survival rate in the intervention group (P = 0.08). Additionally, there was no significant difference in mortality before final discharge.

Treatment with perampanel did not induce any adverse reactions of particular concern. There was one case of ventilator-associated pneumonia in the intervention group, one case of cerebral hemorrhage, and one case of alveolar hemorrhage in the non-intervention group (Table 3). It is unknown whether this was related to the administration of perampanel.

**Table 3:** Outcome and harmful events PER: Perampanel; ICU: intensive care unit; CPC: Cerebral Performance Category Neurological outcomes were not significantly different between the two groups at ICU discharge, but neurological outcomes were significantly better in the perampanel group at hospital discharge.

	PER+(n=33)	PER-(n=33)	p-value
outcome			
primary outcome (ICU discharge)			
CPC score 1-2（number）	16	9	0.12
ICU days（number of survivors）	12.2±4.5	15.3±10.5	0.34
mortality（number of death）	8	11	0.59
ventilator period (days of survivor)	10.8±4.4	17.9±22	0.18
secondary outcome (Hospital discharge)			
CPC score 1-2 (number)	19	9	0.01
Hospitalization days（number of survivors）	36.1 ±33.0	32.5±30.8	0.91
mortality（total number of death）	12	20	0.08
Death after withdrawal of life-sustaining treatment (number)	11	19	0.08
Death, other cause (number)	1	0	1
Harmful events			
bleeding	0	2	0.49
blain	0	1	1
alveolar	0	1	1
ventilator-associated pneumonia	1	0	1
multiple organ failure	0	1	1

Other comparisons of sedatives and antiepileptic drugs showed that propofol was used more frequently in the intervention group; however, there were no significant differences in the use of other sedatives (Table 4).

**Table 4:** Anticonvulsant and sedation PER: Perampanel The perampanel group had more patients receiving propofol, but there were no other differences.

	PER+(n=33)	PER-(n=33)	p-value
Anticonvulsant			
Levetiracetam	32	29	0.36
Lacosamide	3	4	1
clonazepam	1	0	1
Others	2	1	1
Sedation			
Propofol	31	24	0.04
Midazorame	19	21	0.8
Dexmedetomidine	5	4	1
Thiopental	1	1	1

## Discussion

Brain injury associated with cerebral ischemia caused by cardiac arrest can result in primary or secondary damage. Primary injury results from the cessation of aerobic reactions in the brain and the promotion of anaerobic reactions, resulting in the progression of acidosis, an increase in extracellular potassium ion concentration, a decrease in sodium ions, and a rapid intracellular influx of calcium ions, which induces glutamate release. Excess glutamate binds to N-methyl-D-aspartate (NMDA) and AMPA receptors, causing further and sustained intracellular calcium ion influx, resulting in mitochondrial dysfunction and cell death [17-19]. The secondary injury occurs following the primary injury. Reperfusion injury is believed to occur after the blood flow to the brain resumes, releasing free radicals and endothelial dysfunction.

Additionally, microcirculatory disturbances are believed to exacerbate cellular damage. Primary brain injury occurs before resuscitation. Secondary brain damage should be controlled during neurointensive care after ROSC. Perampanel, an antiepileptic drug, exerts its inhibitory anticonvulsant effects by exhibiting a competitive relationship with glutamate in binding with AMPA receptors in the excitatory nervous system. These results show that early post-resuscitation administration of perampanel significantly improved neurological outcomes, possibly inhibiting AMPA receptors. This may have prevented the intracellular influx of calcium ions and thus prevented brain cell death. In addition, perampanel has been reported to significantly suppress microglial activation, inflammatory cytokine expression, and oxidative stress in a cerebral infarction model by inhibiting infarct expansion, suppressing motor function decline, and suppressing secondary neuronal damage in the chronic phase; however, only in animal studies. In humans, it inhibits neuronal apoptosis following head trauma and cerebrovascular barrier damage following subarachnoid hemorrhage [20]. Thus, the competitive inhibitory effect of perampanel on AMPA receptors may reduce secondary brain damage following ROSC.

Although the initial ECG, presence of witnesses, and bystander cardiopulmonary resuscitation were considered important factors in neurological outcomes from cardiopulmonary arrest to resuscitation, there was no significant difference between the two groups in this study. The two groups had no significant differences in the rCAST or severity scores. There were no clear differences between the two groups regarding the cause of cardiopulmonary arrest (Table 1). There were no significant differences in blood draws of items considered indicators of neurological outcome (Table 2) and no significant differences in the GWR based on head computed tomography scans. Although there were no differences in the factors from prehospital to ROSC, the neurological outcome at discharge from the hospital was significantly better in the intervention group than in the non-intervention group, suggesting that early administration of perampanel after ROSC may improve neurological outcomes. There was no difference in the outcomes at ICU discharge; however, there was a difference in the long-term outcomes at hospital discharge. There have been reports of late awakenings after resuscitation, discharge from the ICU, and after a period of >1 month [21], so the final neurological outcome may not be determined by a short ICU stay of an average of 14 days and long-term follow-up is necessary. In fact, in our study, three patients in the intervention group improved CPC scores after discharge from the ICU and had good outcomes. However, in the non-intervention group, there was no improvement in the outcomes after discharge from the ICU. There have been reports of restoration of myelination following the use of perampanel [22], which may contribute to long-term neuronal recovery. In addition to temperature management therapy, the results of this study suggest that drugs may reduce secondary brain damage that occurs after ROSC and may reduce the progression of hypoxic-ischemic encephalopathy. To achieve this, early intervention after ROSC is necessary to reduce secondary brain damage, and early post-resuscitation administration of perampanel may reduce secondary brain damage and improve neurological outcomes. There were also four patients in the non-intervention group in whom perampanel was administered more than 72 hours after resuscitation for refractory seizure control. However, none of the four patients had improved neurological outcomes. This suggests that the administration of perampanel after 72 h does not prevent secondary brain damage or inhibit the progression of hypoxic-ischemic encephalopathy that begins after ROSC, other than its antiepileptic effect and that early administration of perampanel after ROSC is necessary to improve neurological outcomes. EEG findings have been reported as poor neurological outcomes in patients with highly malignant EEG (16). In this study, in the intervention group, eight of 19 patients with the HM pattern on the first EEG within 24 h had a good outcome; in contrast, all patients with the HM pattern in the non-intervention group had a poor outcome (table 5). Although there was no significant difference, these results suggest that early post-ROSC administration of perampanel in the HM group may prevent the progression of hypoxic-ischemic encephalopathy and improve neurological outcomes. Levetiracetam was used in most cases, and the dose of levetiracetam was increased, or other antiepileptic drugs were added based on EEG results and seizure symptoms. Levetiracetam was administered in most cases in both groups, with no significant difference. Additional antiepileptic drugs were administered based on the second EEG findings or seizure symptoms and were added more than 72 hours after resuscitation. Although there are reports of no improvement in neurological outcomes when antiepileptic drugs are administered for HMEEG [a post-ROSC EEG finding [23]], perampanel was not used in this report. The results of the present study suggest that the effects of perampanel may have been exerted by early administration, which may have reduced secondary brain injury.

**Table 5:** Electroencephalographic findings EEG: electroencephalographyic; PER: Perampanel; CPC: Cerebral Performance Category EEG was accurately measured in 31 patients in the perampanel group and 31 in the non-treated group. There was a trend toward improved EEG findings by the time of discharge in the perampanel-treated group.

EEG(total number)	PER＋(n=31)	PER－(n=31)	p-value
Highly malignant EEG (number)	19	19	1
Suppressed Background (number)	8	10	0.78
Suppressed Background with continuous periodic discharges (number)	5	3	0.71
Burst-Suppression Background (number)	5	6	1
non-highly malignant EEG (number)	12	12	1
CPC1.2	PER＋(19)	PER－(8)	
Highly malignant EEG (number)	8	0	0.06
Suppressed Background (number)	2	0	1
Suppressed Background with continuous periodic discharges (number)	2	0	1
Burst-Supression Background (number)	4	0	0.29
non-highly malignant EEG(number)	11	8	0.06

Limitations

The current study found significant differences in neurological outcomes at discharge despite no differences in background. However, the non-intervention group used historical controls and was not a randomized control trial (RCT). Therefore, this single-center study may have been biased. In addition, the total number of patients in the two groups was 66, a small sample size; therefore, RCTs at other centers should be conducted in the future.

## Conclusions

The mainstay of treatment for post-ROSC hypoxic-ischemic encephalopathy has been temperature control therapy; however, there are various reports on the temperature setting and cooling period for temperature control therapy, and there is currently no consensus. There are a few reports on drug treatment for post-resuscitation encephalopathy. Although there are many reports of antiepileptic drug administration for abnormal EEG after resuscitation, there are no reports of favorable outcomes. Considering the mechanism of hypoxic-ischemic encephalopathy, perampanel differs from other antiepileptic drugs that inhibit sodium ion channels because it competes intensively with AMPA receptors and inhibits neuronal cell death, suggesting that perampanel treatment may contribute to improved neurological outcomes. This study's results also suggest that administering perampanel immediately after ROSC may inhibit secondary brain damage and improve the neurological outcomes of hypoxic-ischemic encephalopathy.
